# Secondary Brown Carbon Formed by a Microreactor of a Levitated Aqueous Fe (III) Droplet with Fumaric Acid

**DOI:** 10.1021/acsphotonics.5c01691

**Published:** 2025-12-25

**Authors:** Gema Sánchez-Jiménez, Hind A. Al-Abadleh, Daniel Pérez-Ramírez, Lucas Alados-Arboledas, Francisco José Olmo-Reyes, Antonio Valenzuela

**Affiliations:** † Andalusian Institute for Earth System Research (IISTA-CEAMA), 16741 University of Granada, Granada 18006, Spain; ‡ Department of Applied Physics, University of Granada, Granada 18071, Spain; § Department of Earth, Environmental and Resource Sciences, 12337 University of Texas at El Paso, 500 West University Avenue, El Paso, Texas 79902, United States

**Keywords:** radiative forcing efficiency, aerosol aging, brown carbon, single levitated microdroplets, mie theory, spectral complex refractive index

## Abstract

This study investigates the influence of fumaric acid on the optical and microphysical properties of aqueous FeCl_3_ microdroplets and how aging affects them. This process replicates a pathway for brown carbon (BrC) formation in the atmosphere. The experiment combines a Paul electrodynamic trap (PET), which captures a single particle, and a dual-wavelength cavity ring-down spectroscopy (CRDS) system. Initially, measurements were conducted under controlled humidity cycling, obtaining the particle phase function at a 532 nm wavelength. Retrievals reveal an irreversible increase in particle radius and complex refractive index (*m*_λ_ = *n*_λ_ + *ik*·_λ_) after a dehydration–hydration cycle. The second part involves measuring a single particle trapped from the FeCl_3_ + fumaric acid solution after 24 h in darkness. Instrumental flexibility enabled complementary measurements of the particle phase function at 473, 532, and 660 nm wavelengths and the extinction cross-section (σ_ext,λ_) at 405 and 532 nm wavelengths. The most significant result was the retrieval of multiwavelength *m*_λ_, revealing a strong spectral dependence of *k*_λ_, which decreased from 0.014 at 405 nm to 0.000 at 660 mn. Radiative effects were evaluated and compared with other oxidation pathways of fresh biomass tar proxies, highlighting the need for precise BrC characterization in climate models, particularly in the UV range.

## Graphical abstract

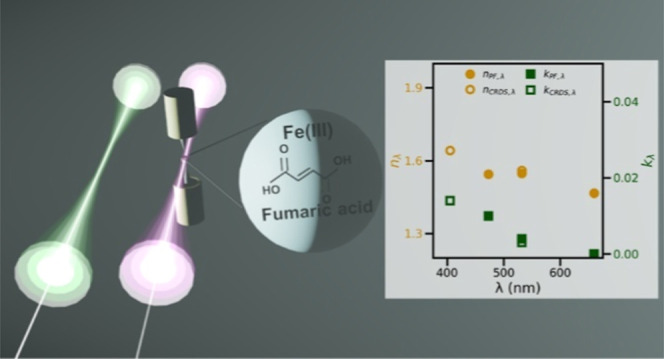

## Introduction

Atmospheric organic aerosols (OA) constitute between 20 and 50% of global aerosols, reflecting the complex interaction between natural and anthropogenic processes.^1,2^ The composition of these compounds is diverse, encompassing a range of chemical species, ranging from simple hydrocarbons to highly oxidized compounds. Primary organic aerosols (POA) are emitted directly, while secondary organic aerosols (SOA) are formed through the oxidation of volatile and semivolatile organic compounds (VOCs).^3^

It is evident that biomass burning events represent a significant source of POA and VOC emissions, thereby introducing intermediates that can complicate the OA landscape. Heterogeneous processes in the particulate and gaseous phases play a key role in SOA formation by accreting low-volatility species.^4–6^ Dust particles composed of different elements are examples of heterogeneous processes. One such element, iron, which is the fourth most abundant element in the Earth’s crust, is present in dust from both natural and anthropogenic sources. In the context of long-distance transportation, these particles undergo atmospheric processing, thereby interacting with organic gases, including those emanating from biomass burning. This interaction subsequently leads to the formation of SOA.^7,8^

In addition to photochemical and free-radical-driven processes, the field of SOA research has recently witnessed a surge of interest in redox chemistry in the absence of light.^9^ In previous research, the role of iron (Fe) in catalyzing reactions under conditions of high humidity has been demonstrated.^10^ These conditions result in the formation of water-insoluble and soluble brown carbon (BrC). The utilization of advanced optical microscopy was instrumental in unveiling the dynamic morphological changes occurring in aqueous microdroplets encompassing unsaturated C4–C6 dicarboxylic acids (for instance, fumaric acid) and Fe (III) within aged SOAs. Recent laboratory investigations have confirmed that this Fe (III)-catalyzed reaction with fumaric acid leads to the formation of conjugated organometallic polymers that exhibit broadband absorption in the near-UV and visible spectrum, a hallmark of atmospherically relevant BrC.^10,11^ The hypothesis is that the formation of these iron-organic complexes occurs under acidic conditions prevalent in cloud droplets, fog, and deliquescent aerosols, where soluble iron (e.g., FeCl_3_) is present from dust or combustion sources.^10–12^ It is important to note that the reaction occurs even in the presence of atmospheric ligands such as sulfate or nitrate. This suggests that it could be a significant contributor to the aqueous-phase BrC formation.

The FeCl_3_–fumaric acid system functions as a chemically simplified but atmospherically relevant proxy to replicate one of the proposed aqueous-phase pathways for BrC formation. The resulting polymeric products are also morphologically and optically distinct, often forming amorphous, poorly soluble surface films on droplets, with enhanced absorption near 370 nm, consistent with BrC’s spectroscopic features.^10,11^ Their hydrophobic and insoluble nature also suggests implications for droplet phase separation and cloud condensation nuclei (CCN) activity.^13^

The complex chemical composition of atmospheric aerosol particles exerts a significant influence on their physical properties, including their morphology and mixing state.^14^ To investigate these aspects, the reaction between fumaric acid and Fe (III) was monitored in micrometer-sized droplets under a controlled humid air flow (96–100% relative humidity (RH)) using optical spectroscopy at the University of British Columbia. The results obtained from this study revealed the formation of an Fe-polyfumarate at the air–aqueous interface. Furthermore, the occurrence of visible movement of the polymer on the droplet surface was observed, indicating a core–shell morphology. The findings emphasize significant alterations in the morphology, mixing state, and chemical composition of droplets resulting from the presence of insoluble iron-based products, which consequently impact droplet chemistry.^15^ As asserted by Lang-Yona,^16^ SOA and BrC, formed in aqueous environments such as from deliquescent particles and cloud droplets, constitute intricate mixtures of dissolved molecules. These substances undergo substantial transformations during the processes of evaporation and hydration. Recent studies that employed single particle levitation techniques have yielded novel insights into the dynamics and optical properties of SOA. Evaporation, for instance, has been demonstrated to be influenced by the solubility of SOA components and insoluble inclusions. This phenomenon gives rise to the formation of diverse particle states, including homogeneous, trapped, and core–shell configurations. It has been observed that these states have a significant effect on optical properties and radiation interactions.^16^

The phenomenon of Earth-atmosphere radiative forcing is found to be directly influenced by SOA, given the capacity of these particles to scatter and absorb both shortwave and longwave radiation, thereby engendering a mixture of warming and cooling effects.^7^ It has been demonstrated that SOA particles have the capacity to influence the concentration of CCN, thereby modulating the process of cloud condensation.^17^ To accurately comprehend the radiative effects in question, it is imperative to undertake a thorough analysis of the optical properties of SOA. These properties have been shown to depend on the particle radius, *r*, and the spectral complex refractive index, *m*_λ_ = *n*_λ_ + *i*·*k*_λ_. The real part of the index of refraction, *n*_λ_, is related to the phase velocity of light propagating through the medium, directly linked to the scattering of the medium, while the imaginary part, *k*_λ_, corresponds to the attenuation of the wave due to absorption within the medium.^18^ The chemical aging of aerosol particles, facilitated by multiphase reactions in bulk, on surfaces, or in cloud droplets, ultimately alters these optical properties and redefines the radiative behavior of SOA.^19,20^

However, it should be noted that bulk-phase or ensemble-averaged studies frequently obscure the physicochemical diversity of individual particles and limit our ability to resolve subtle aging processes such as morphology transitions, the formation of insoluble coatings, or changes in water uptake. Conversely, single-particle analysis facilitates the real-time observation of these transformations with high temporal and spatial resolution, thereby providing critical insight into the mechanisms that govern the evolution of SOA optical properties.

The present study employs a pioneering experimental platform that integrates a Paul electrodynamic trap (PET) with a dual-wavelength cavity ring-down spectroscopy (CRDS) system, enabling precise measurement of the *m*_λ_ and *r* of a single levitated particle under RH relevant to the atmosphere. This system represents a significant methodological advancement in the field of aerosol research, thereby facilitating the detection of dynamic changes that are otherwise inaccessible to conventional bulk techniques. The PET–CRDS configuration also reduces particle-to-particle variability by holding a single particle static in the trap while translating transversely relative to two independent cavities, allowing for the simultaneous acquisition of the phase function at 473, 532, and 660 nm wavelengths and extinction cross section (σ_ext,λ_) measurements at 405 and 532 nm wavelengths. The employment of an inversion algorithm founded upon Mie theory facilitates the retrieval of the *r*, in conjunction with the *n*_λ_ and *k*_λ,_ thereby enabling the temporal tracking of Fe (III)-catalyzed reactions with fumaric acid. Despite the unfeasibility of direct comparison between single-particle measurements and values derived from ensemble methods, such as flow-cell or chamber studies, is not possible, the complementarity of the two approaches is emphasized.

While bulk measurements are capable of averaging over diverse particle populations, the approach adopted here can isolate well-defined particle states, thereby enabling the capture of transient processes such as surface polymerization or phase separation. This distinction is critical to understanding the formation of optically active BrC and its role in atmospheric radiative processes.

In summary, the present study introduces a novel single-particle analytical approach and provides new insights into BrC formation via iron-organic chemistry. This challenges previous paradigms and bridges laboratory studies with real atmospheric microenvironments.

## Results and Discussion

### Optical and Microphysical Properties of Dehydrated and Hydrated FeCl_3_ and Fumaric Acid Microdroplets

We evaluated the temporal evolution of the optical and microphysical properties of an FeCl_3_–fumaric acid microdroplet exposed to a full hygroscopic cycle. Figure 1 shows the temporal evolution of a droplet of: (a) %RH, (b) the Pearson correlation coefficient (C), (c) the fitting *r* derived from Mie theory, and (d,e) the real *n*_PF,532_and imaginary *k*_PF,532_parts of the refractive index at a wavelength of 532 nm. The subscript PF denotes that these refractive index components were retrieved from fits to the phase function. The experiment started approximately 300 s after adding solid FeCl_3_ to the fumaric acid solution, which was done under dark conditions. Measurements were conducted within a controlled environment with the initial RH set to 80% (Figure 1a, blue shaded region). This was maintained for a period of 1380 s, after which subsequent modifications to the RH were made. At around the 1400th second, the droplet underwent controlled dehydration to approximately 50% RH, followed by rehydration to ∼78% RH. This hydration cycle was completed around the 2730th second, after which the RH remained stable until the end of the experiment (Figure 1, yellow shaded regions).

**Figure 1:**
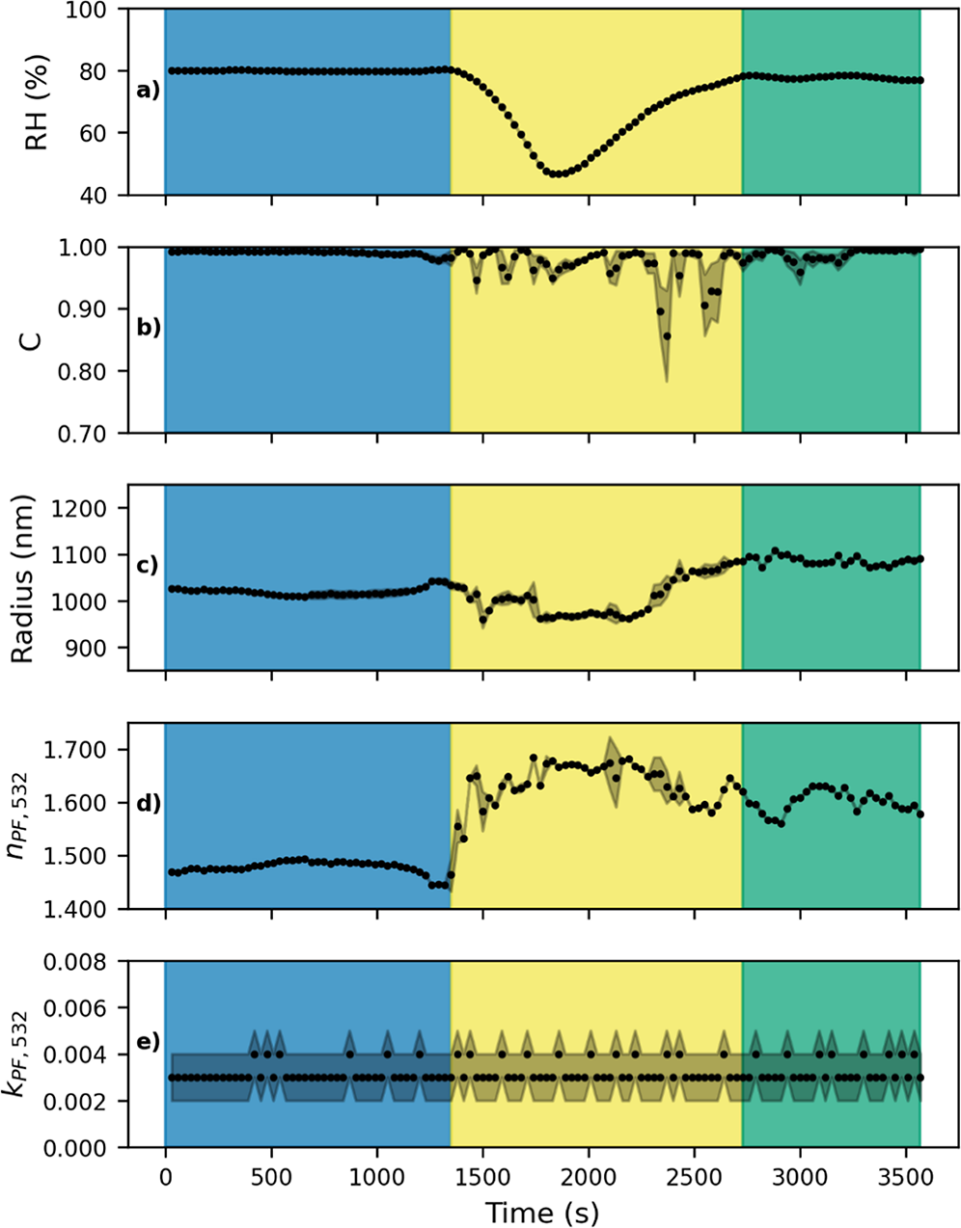
Representative evolution of the optical and microphysical properties of a microdroplet of FeCl_3_ with fumaric acid. The shaded area adjacent to the dots denotes the standard deviation. The plots showing: (a) the values in measured %RH, (b) Pearson’s correlation coefficient (C), (c) fitting *r* from Mie theory, (d) *n*_PF,532,_ and (e) *k*_PF,532_ with increasing reaction time. The shaded regions correspond to the initial RH (blue), the dehydration and hydration cycle (yellow), and the final RH (green).

The phase function was recorded at one second intervals to monitor changes in particle properties. For the purposes of clarity, the parameters shown in Figure 1 were calculated by averaging 30 consecutive phase functions, as no significant variations in optical and microphysical properties were observed within these intervals. The quality of fit was assessed using C, which approached unity, as demonstrated in Figure 1b. During the first 1380 s of the experiment, *r* remained approximately constant with mean values of 1042 ± 2 nm (Figure 1c). In a similar manner, the retrieved *n*_PF,532_ of 1.442 ± 0.004, remained constant at that initial stage, whereas *k*_PF,532_ showed values between 0.001 and 0.004 (Figure 1d,e). Retrieved optical and microphysical properties showed significant modifications for *n*_PF,532_ (reaching values close to 1.670), while *k*_PF,532_ remained within the range of values. Particle radii decreased slightly during dehydration and increased during rehydration.

The key result of the experiment is observed when returning to the initial RH values (Figure 1, green shaded region) because the increase in r (1083 ± 8 nm) and *n*PF_,532_ (1.602 ± 0.013) remains even when we return to the initial RH values. The high C, which exceeds 0.996 at this final stage, provides a high degree of reliability for the optical and microphysical properties obtained. The *n*PF_,532_ value obtained is consistent with the findings reported in ref 19, which employed broadband cavity enhanced spectroscopy to measure the *n*_λ_ and *k*_λ_ of NH_3_-aged SOA (360–420 nm). They observed an increase in *n*_360–420_ for α-pinene SOA from 1.50 to 1.57 after 1.5 h at 1.9 ppm of NH_3_, while *k*_360–420_ remained below 0.001. In contrast, limonene and α-humulene SOA exhibited negligible changes in *n*_360–420_ but slight increases in *k*_360–420_ (0.032 and 0.029, respectively). These findings underscore the variable optical response of organic aerosols to chemical aging. All of the droplets analyzed in our study exhibited initial radii between 900 and 1200 nm.

For comparison, Figure S1 presents data from a control experiment involving a levitated aqueous microdroplet containing only FeCl_3_ and no fumaric acid, recorded over 4200 s. Figure S1 demonstrates the absence of any change in the *r*, *n*_532,_ or *k*_532_. These parameters exhibited stability and consistency with predictions from the Lorentz–Mie theory. C remained close to unity throughout the experiment, validating the applicability of Lorentz–Mie theory for the retrievals of optical and microphysical properties of nonreactive FeCl_3_ droplets, which are well-characterized as spherical and homogeneous.

In contrast, the experimental data in Figure 1 demonstrate the transformative influence of fumaric acid, a reactive dicarboxylic compound, on the physicochemical properties of a microdroplet. Unlike the control experiment, the presence of fumaric acid induced significant changes in the droplet size and composition. These findings highlight the impact of hygroscopic cycles on the optical and microphysical properties of aqueous microdroplets containing FeCl_3_ and fumaric acid. The irreversible increase in *r* and *n*_532_ following the dehydration–hydration cycle, despite the return of RH to almost initial levels, highlights the intricate interplay between chemical composition, water content, and optical behavior. The employment of single particle phase function fitting with Lorenz–Mie theory has enabled the study to achieve a level of temporal resolution in tracking these transformations that is unparalleled in the existing literature. This has led to the identification of dynamic processes that would have otherwise remained undetected by using ensemble particles. The consistency of these results with those of previous studies on chemically aged organic aerosols validates the methodology, while the distinct optical response observed here emphasizes the unique physicochemical evolution of the Fe (III)-fumaric acid system under varying humidity conditions.^21^ These insights are of crucial importance for the improvement of atmospheric models and the advancement of our understanding of aerosol–cloud interactions, and they demonstrate the effectiveness of this approach in uncovering intricate aerosol transformation mechanisms with high temporal accuracy.

### Wavelength-Dependent on Complex Refractive Index of FeCl_3_ with Fumaric Acid Microdroplet

To evaluate the impact of chemical aging on the optical properties, we retrieved the wavelength-dependent complex refractive index of a FeCl_3_–fumaric acid microdroplet aged for 24 h under dark conditions. The combined use of phase function and dual-wavelength CRDS measurements enabled the determination of both *n*_λ_ and *k*_λ_, as well as the *r*. The results shown in Figures 2 and 4 were obtained from the same representative droplet, chosen to exemplify the characteristic optical response observed across all replicates, which had radii in the range of 900–1200 nm. For clarity, the real and imaginary parts of the refractive index are denoted with subscripts PF and CRDS, respectively, in order to distinguish between fittings derived from the phase function (*n*_PF_,_λ_, *k*_PF,λ_) and those obtained from CRDS measurements (*n*_CRDS_,_λ_, *k*_CRDS_,_λ_), respectively.

**Figure 2:**
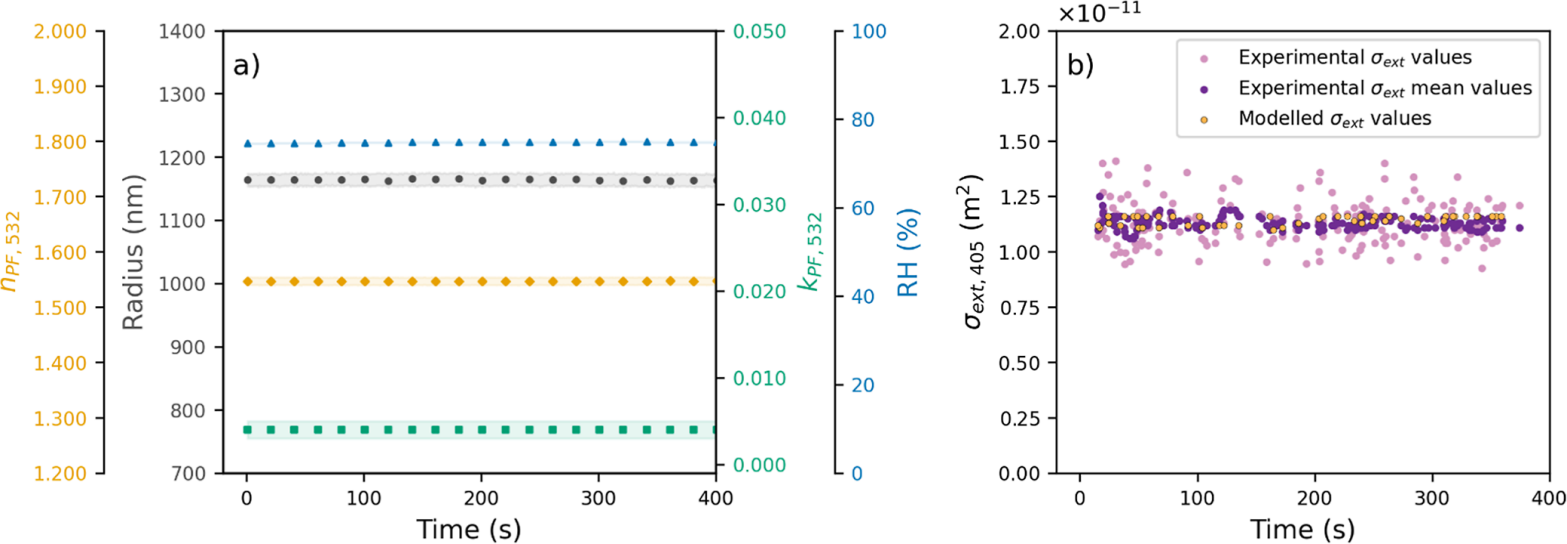
(a) Evolution of the retrieved parameters (*n*_PF_,_532_, *k*_PF_,_532_, and *r*) from particle phase function measurements at 532 nm, where the shaded area denotes the standard deviation and (b) experimental mean values (dark purple), experimental values (clear purple), and modeled values (yellow) of σ_ext_,_405_.

**Figure 3:**
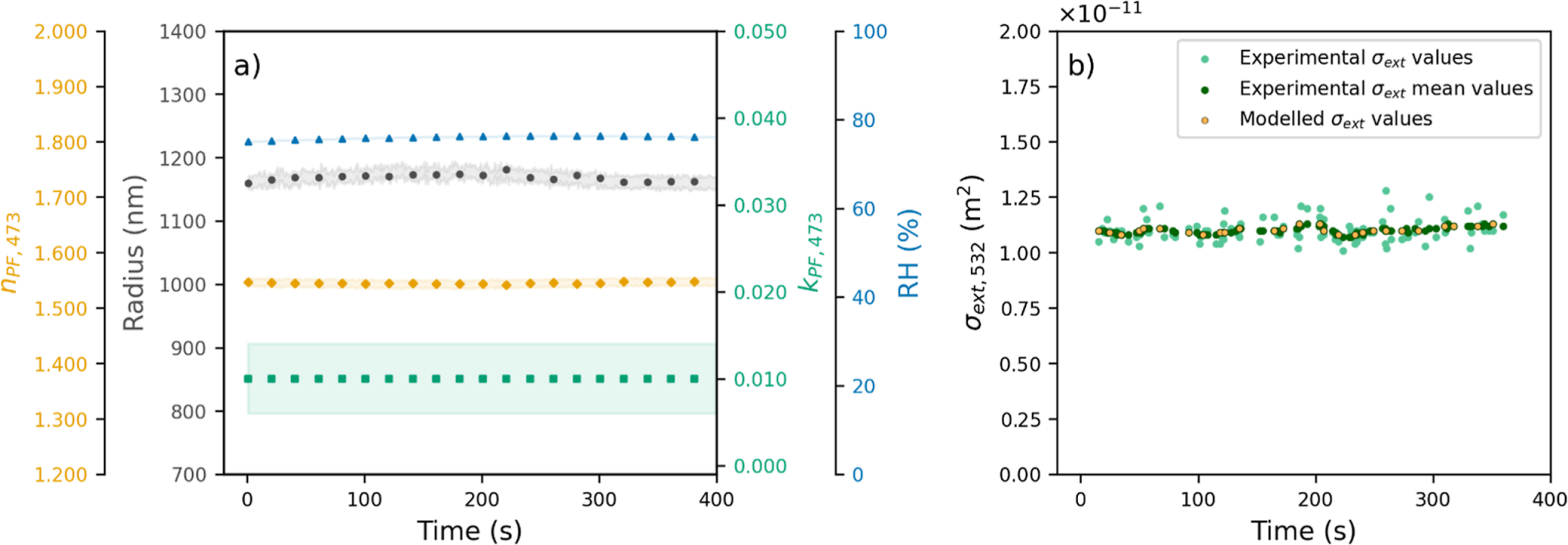
(a) Evolution of the retrieved parameters (*n*_PF_,_473_, *k*_PF_,_473_, and *r*) from particle phase function measurements at a wavelength of 473 nm, where the shaded area denotes the standard deviation, (b) experimental mean values (dark green), experimental values (green), and modeled values (yellow) of σ_ext_,_532_.

**Figure 4:**
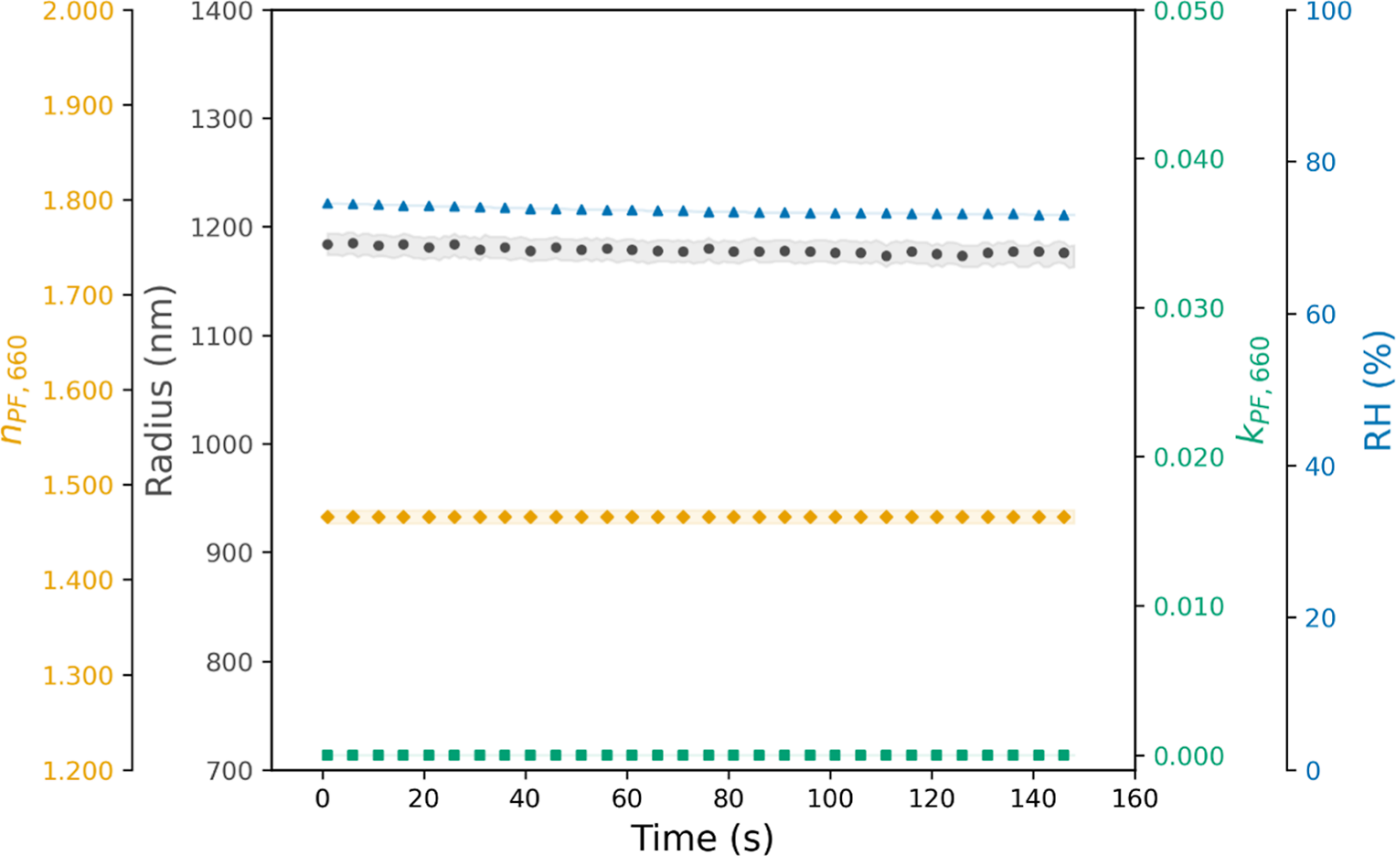
Evolution of the retrieved parameters (*n*_PF_,_660_, *k*_PF_,_660_, and *r*) from particle phase function measurements at 660 nm, where the shaded area denotes the standard deviation.

The experiment started by placing the PET in the 405-CRDS system, where the phase functions at a wavelength of 532 nm, and the ring-down time at 405 nm wavelength (τ_405_) measurements were collected for approximately 550 s (Figure S2a in Supporting Information). Figure 2a represents the results of fitting the experimental phase functions to a theoretical library, allowing the retrieval of *n*_PF_,_532_, *k*_PF_,_532_, and *r*. Furthermore, the RH was maintained at a consistent level throughout the experiment (RH = 74.76 ± 0.09%) to prevent exposure to processes such as evaporation or hydration. Therefore, the optical and microphysical properties are expected to remain largely stable. This is consistent with the approximately constant retrieved values illustrated in Figure 2a: *r* = 1164 ± 4 nm, *n*_PF_,_532_ = 1.547 ± 0.005, and *k*_PF_,_532_ = 0.004 ± 0.001. The shaded area in the figure corresponds to the standard deviation. Figure 2b shows the calculated extinction cross sections at 405 nm wavelength (σ_ext_,_405_) obtained from τ_405_ measurements and reveals a consistent mean σ_ext_,_405_ value of (1.12 ± 0.03) × 10^–11^m^2^. No significant variations in the mean σ_ext_,_405_ values were observed, confirming the absence of changes in the optical and microphysical properties. The combination of experimental measurements of σ_ext_,_405_ with simultaneous and independent data of *n*_PF_,_532_, *k*_PF_,_532,_ and *r*, along with their associated uncertainties, derived from phase function measurements, provides a more comprehensive retrieval scheme for particle optical properties at 405 nm wavelength. This has a substantial impact on the range of potential retrieval possibilities for reproducing experimental σ_ext_,_405_. This enables a more feasible and accurate retrieval of *n*_CRDS_,_405_ and *k*_CRDS_,_405_. The values obtained were 1.642 ± 0.002 and 0.014 ± 0.004, respectively.

Subsequently, the PET was relocated to the second 532-CRDS system, where phase functions at 473 nm and the ring-down time at 532 nm wavelength (τ_532_) measurements were obtained for approximately 370 s (see Figure S2b in the Supporting Information). The RH was again kept constant at a mean value of 75.95 ± 0.09%. Figure 3a shows the retrieved values of *n*_PF_,_473_, *k*_PF_,_473_, and *r* computed by fitting the experimental particle phase functions to a Lorentz–Mie theoretical library of phase functions. The mean values retrieved were *n*_PF_,_473_ = 1.544 ± 0.002, *k*_PF_,_473_ = 0.010 ± 0.004, and *r* = 1168 ± 7 nm. The *r* value obtained from the fit is consistent with those obtained from phase functions at a wavelength of 532 nm (Figure 2a), which further validates the reliability of our data and the accuracy of the refractive index values provided. However, there are significant differences in *n*_PF_,_473_ and *k*_PF_,_473_ compared with values at 532 nm wavelength, with the increase being more pronounced for the imaginary part of the refractive index.

Figure 3b represents the calculated extinction cross sections at 532 nm wavelength (σ_ext_,_532_) from τ_532_ measurements. The σ_ext_,_532_ exhibits a consistent mean value of (1.13 ± 0.04) × 10^–11^m^2^, remaining stable throughout the experiment. This finding is consistent with observations made using the 405-CRDS system, despite the wavelength dependence of the extinction cross section. Moreover, the fitting parameters from the phase function at a wavelength of 473 nm and the experimental σ_ext_,_532_ values were used to model the σ_ext_,_532_ and consequently retrieve *n*_CRDS_,_532_ and *k*_CRDS_,_532_ (Figure 3b). The mean values obtained for *n*_CRDS_,_532_ and *k*_CRDS_,_532_ were 1.560 ± 0.008 and 0.003 ± 0.001, respectively. These values generally agree with those previously obtained at 532 nm for the 405-CRDS system configurations, with differences within the standard deviations.

Finally, the 405-CRDS system is illuminated by a laser of wavelength 660 nm, enabling measurements of the particle phase function at 660 nm wavelength to be taken. The measurement time was approximately 160 s, and RH was maintained at a constant mean value of 73.55 ± 0.44%. Again, fitting experimental phase functions to Mie theoretical models allowed us to retrieve *n*_PF_,_660_, *k*_PF_,_660,_ and *r*, with the retrieved values over time shown in Figure 4. The mean retrieved *r* value was 1178 ± 6 nm, while *n*_PF_,_660_ had a mean value of 1.466 ± 0.001, with negligible absorption (*k*_PF_,_660_ = 0.000).

The retrieved particle radii using the three data sets with phase function measurements (473, 532, and 660 nm) are basically identical for the different wavelengths; differences are within the standard deviations and give consistency to the results of our experiment. Moreover, this consistency gives reliability to retrieved *m*_λ_ values with measurements of extinction cross section (wavelengths of 405 and 532 nm for the 405-CRDS and 532-CRDS configurations, respectively). Figure 5 presents retrieved *n*_λ_ and *k*_λ_ as a function of wavelength, showing excellent agreement between the CRDS and PF methodologies at a wavelength of 532 nm, with differences remaining within the respective methodological uncertainties. However, the most important result is the sharp spectral dependence in *k*_λ_ with values at a wavelength of 405 nm (0.014 ± 0.004), 1 order of magnitude higher than those at 532 nm wavelength (0.004 ± 0.001), indicating enhanced absorption in the ultraviolet range. For *n*_λ,_ there is also an important spectral dependence (1.642 at 405 nm and 1.466 at 660 nm), but it is less pronounced for the imaginary part.

**Figure 5:**
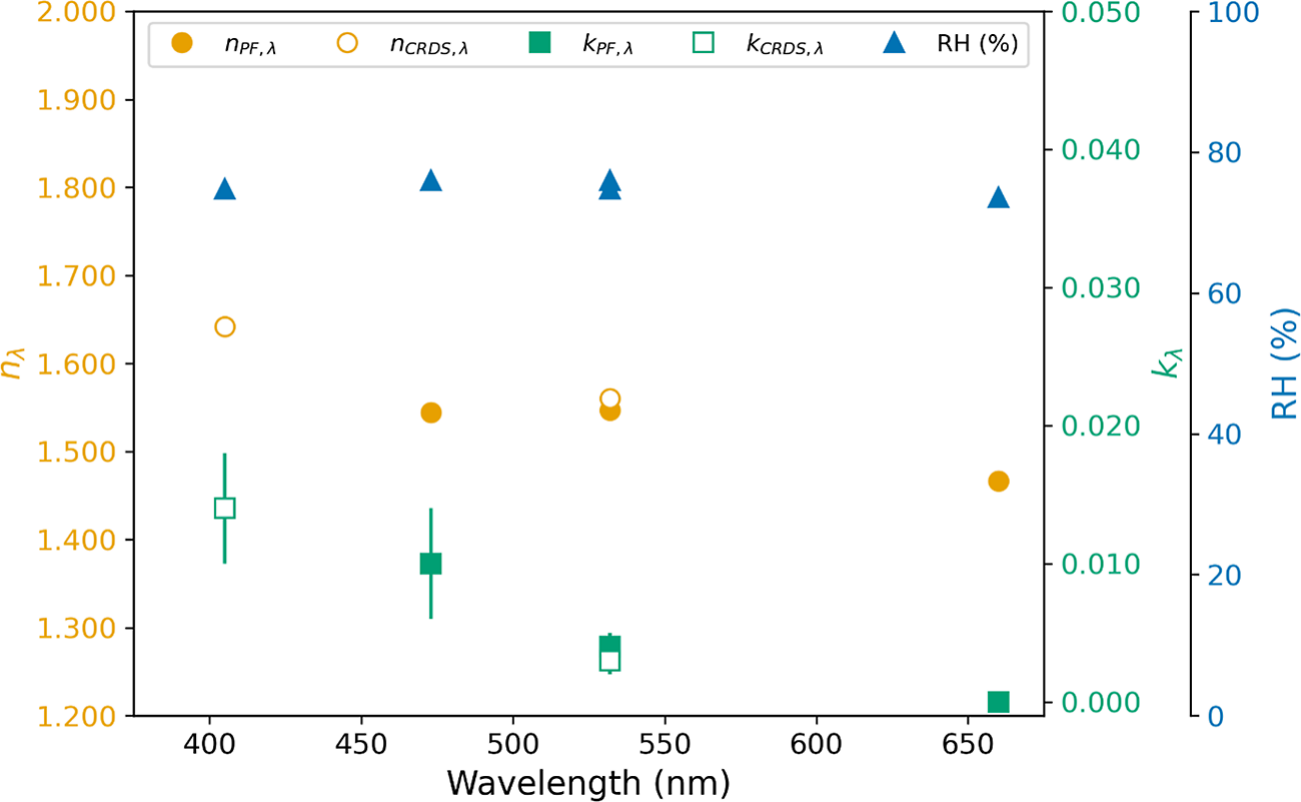
Retrieved *n*_λ_ and *k*_λ_ as a function of wavelength. Subscript CRDS indicates values obtained from particle extinction cross-section measurements, while PF subscripts refer to values derived from phase function retrievals. The error bars correspond to standard deviations. This representation allows for a direct comparison between optical properties retrieved by different methods and highlights the wavelength dependence of the refractive index components.

Overall, the results in Figure 5 demonstrate consistency with previously reported refractive index values for SOA derived from α-pinene via various formation pathways, including ozonolysis, OH oxidation, and photo-oxidation in the presence of NOx: While Nakayamal^22^ reported an *n*_532_ of 1.411 ± 0.021 with an imaginary component *k*_532_ of 0.000 (with an uncertainty range of +0.025 to −0.000), Lambe^23^ found an *n*_405_ of 1.51 ± 0.02 and a *k*_405_ of less than 0.001 for OH oxidation. Similarly, Barkey^24^ obtained an *n*_670_ of 1.42 ± 0.02 with a *k*_670_ of zero under photochemical oxidation conditions in the presence of NOx.

Despite the differences in the mechanisms of formation, the complex refractive index values that were retrieved remained within the range that has been reported in the literature for the considered wavelength spectrum. This finding serves to reinforce the robustness of the results obtained in this study. The central innovation of this study lies in the capacity to measure the spectral complex refractive index of a single particle while concurrently controlling for diverse phases of BrC evolution. This capability enhances the relevance of the approach adopted and expands the possibilities for characterizing the optical properties of BrC.

While the present study employs a single-particle methodology, the comparison with ensemble-averaged measurements from the literature is both scientifically justified and highly informative. It is evident that ensemble techniques inherently average over polydisperse populations and are thus incapable of resolving the heterogeneity of individual particle properties, including but not limited to morphology, internal mixing, and surface-phase reactions. In contrast, the present measurements can isolate well-characterized particles and capture temporal evolution under controlled humidity. Therefore, notwithstanding the differences in methodology, the substantial congruence between our single-particle data and bulk studies furnishes a valuable linkage that connects microscale transformations to population-level trends. This complementarity helps to contextualize our findings within the broader body of atmospheric aerosol research and underscores the atmospheric relevance of the Fe–fumarate system as a potential BrC source.

The main novelty of our innovative experimental setup is that, for the first time, it enables detailed and unambiguous characterization of a single BrC particle formed by the reaction between FeCl_3_ and fumaric acid. The electrodynamic trapping system utilized in this study has been comprehensively characterized in a preceding study,^25^ wherein the phase functions and extinction cross sections of prominent substances, including sodium chloride and 1,2,6-hexanetriol, were measured. In that work, a minimum particle radius of ∼171 nm was detected. These results confirm the system’s sensitivity and its ability to trap particles within the atmospheric accumulation mode (0.1–2 μm diameter). Precise micrometric control facilitates the centering of the particle within the Gaussian mode TEM_00_ of the laser beam, thereby ensuring the reproducibility and stability of measurements of σ_ext_,_λ_. It is therefore concluded that the current experimental configuration is robust and suitable for the inversion-based retrieval of *n*_λ_ and *k*_λ_ from single particles. Moreover, the instrument’s unique design allowed simultaneous and independent measurements from different data sets on the same particle, further increasing result reliability. Finally, comparison with previous studies confirms that our values align with reported ranges, regardless of variations in BrC formation pathways. The findings of this study offer a valuable perspective on the evolution of BrC and its optical relevance in the atmosphere by linking single-particle precision with ensemble-based context.

## Atmospheric Implications

### Radiative Forcing Efficiency Analysis

*k*_λ_ is a dimensionless parameter that serves to quantify the light absorption capacity of a particle. In our analysis, *k*_λ_ exhibits a strong spectral dependence and plays a crucial role. Comparisons with other studies reveal that BrC absorption properties depend on different particle formation processes. Therefore, it is important to estimate the impact of different BrC types on Earth’s radiative forcing to better understand the impact of particle absorption, which has been identified as crucial for reducing uncertainties in aerosols in climate modeling. Specifically, we evaluate four BrC pathways: the one obtained in our study, those reported for fresh biomass burning (wood) tar proxy, and transformations through oxidation with nitrate radicals (NO_3_) and ozone (O_3_) under dark conditions in ref 11.

Table 1 summarizes the wavelength-dependent real and imaginary refractive indices, *n*_λ_ and *k*_λ_, respectively, the single scattering albedo ( ${\mathrm{\omega ̅}}_{\lambda }$ ), the asymmetry parameter (*g*_λ_), and the backscattering fraction (β_λ_)­for the different BrC formation pathways. For the BrC droplets measured in this work, uncertainties in *n*_λ_ and *k*_λ_ were experimentally retrieved and propagated using Monte Carlo simulations, where each iteration performed a full Mie calculation. This approach yields distributions for ${\mathrm{\omega ̅}}_{\lambda }$ , *g*_λ_, β_λ_, and the radiative forcing efficiency (RFE_λ_) (Table 2). In contrast, for biomass burning wood tar aerosol (fresh, O_3_-oxidized, and NO_3_-oxidized), refractive index values were taken from literature sources where only single *n*_λ_ and *k*_λ_ values are reported, without associated variability. Therefore, no uncertainty propagation could be performed for those cases, and only the mean values are included. To simplify comparison and ensure consistency among the four systems, all calculations were conducted by assuming the same particle radius of 1170 nm, which corresponds to the mean droplet size retrieved experimentally in our setup.

**Table 1:** Mean Real and Imaginary Refractive Indices, *n*_λ_ and *k*_λ_, Respectively, and Derived Aerosol Optical Properties $(\overset{®}{\omega_{\lambda }\;},g_{\lambda }\text{and}\;\beta_{\lambda }$ ) for the Mean BrC Values Obtained in this Study are Shown Alongside Those for Biomass Burning Wood tar Aerosol (Fresh, O_3_-Oxidized, and NO_3_-Oxidized)^c^

λ/*nm*		*n*_λ_	*k*_λ_	${\mathrm{\omega ̅}}_{\lambda }$	*g*_λ_	β_λ_
405	O_3_, *w* ^a^	1.635	0.010	0.772	0.837	0.151
	BrC^b^ (5)	1.642 ± 0.008	0.014 ± 0.002	0.719 ± 0.025	0.853 ± 0.009	0.144 ± 0.004
	fresh, *w* ^a^	1.620	0.018	0.661	0.868	0.137
	NO_3_, *w* ^a^	1.592	0.043	0.536	0.914	0.117
473	O_3_, *w* ^a^	1.630	0.005	0.714	0.842	0.210
	BrC^b^ (5)	1.544 ± 0.011	0.010 ± 0.001	0.789 ± 0.015	0.818 ± 0.006	0.159 ± 0.003
	fresh, *w* ^a^	1.610	0.010	0.776	0.794	0.171
	NO_3_, *w* ^a^	1.591	0.038	0.573	0.171	0.124
532	O_3_, *w* ^a^	1.620	0.000	1.000	0.750	0.194
	BrC^b^ (5)	1.547 ± 0.002	0.004 ± 0.001	0.897 ± 0.023	0.750 ± 0.006	0.192 ± 0.003
	fresh, *w* ^a^	1.600	0.001	0.975	0.744	0.195
	NO_3_, *w* ^a^	1.589	0.017	0.713	0.831	0.154
650	O_3_, *w* ^a^	1.590	0.000	1.000	0.683	0.225
	BrC^b^					
	fresh, *w* ^a^	1.570	0.000	1.000	0.730	0.212
	NO_3_, *w* ^a^	1.565	0.000	1.000	0.710	0.202
660	O_3_, *w* ^a^					
	BrC^b^ (5)	1.466 ± 0.002	0.000	1.000	0.796 ± 0.001	0.170 ± 0.001
	fresh, *w* ^a^					
	NO_3_, *w* ^a^					

a Note: these values are given in ref 11.

b Data at wavelengths of 473, 532, and 660 nm were obtained from phase-function measurements, while data at a wavelength of 405 nm were derived from CRDS measurements. The standard deviations of *n*_λ_ and *k*_λ_ were calculated from measurements of 5 individual droplets. The standard deviations of $\overset{®}{\omega_{\lambda }\;},g_{\lambda }\text{and}\beta_{\lambda }$ arise from Monte Carlo error propagation.

c The number in brackets (*n*) represents the number of droplets used to obtain the mean value. Results are shown at wavelengths of 405, 473, 532, 650, and 660 nm.^11^

**Table 2:** RFE_λ_ for Biomass Burning Wood Tar Aerosol (Fresh, O_3_-Oxidised, and NO_3_-Oxidised) and the BrC at 405, 473, 532, 650, and 660 nm Wavelengths

λ/*nm*	${RFE}_{\lambda ,O_{3},W}$ /WAOD^–1^m^–2^	RFE_λ,BrC_/WAOD^–1^m^–2^	RFE_λ,Fresh,_ *_W_*/WAOD^–1^m^–2^	${RFE}_{\lambda ,NO_{3},W}$ /WAOD^–1^m^–2^
405	–2.49	1.56 ± 1.88^a^	5.78	14.89
473	–12.67	–4.41 ± 1.18^a^	–4.53	12.17
532	–19.28	–14.83 ± 1.91^a^	–20.58	1.12
650	–28.95		–24.29	–23.11
660		–19.48 ± 0.02^a^		

a The standard deviations of RFE_λ,BrC_ were obtained through Monte Carlo error propagation.

Table 1 shows that *n*_λ_ for wood tar aerosol resulting from biomass combustion generally has values slightly higher than those of BrC obtained in the present study (maximum differences are 0.04), except for the 405 nm wavelength. For *k*_λ,_ however, all cases show strong spectral dependence on wavelength, ranging from values close to zero at 660 nm (nonabsorbing) to values between 0.01 and 0.043 at 405 nm (high absorption). Similarly, although smaller, high values are observed at 473 nm, while large variability is observed at 532 nm, ranging from values close to zero (nonabsorbing) for O_3_-oxidized samples to 0.017 (high absorption) for NO_3_-oxidized samples. The value for BrC obtained in this study at this wavelength is in the middle (∼0.004, which can be considered as medium absorption). It is therefore evident that the most significant result is the consensus among all studies on the pronounced spectral dependence of *k*_λ_, although there are discrepancies in the intensity of this dependence depending on the mechanism of particle formation. The experimental results of BrC presented in this study support all of these findings by virtue of the uniqueness of measuring the complex refractive index at this wavelength for the same individual particle and with two different approaches at 532 nm.

The remaining optical properties ( $\overset{®}{\omega_{\lambda }\;}$ , *g*_λ_,β_λ_) presented in Table 1 are more sensitive to the varying formation processes, although caution must be exercised due to the internal assumptions made for the size distribution in the computations of these parameters. For ${\mathrm{\omega ̅}}_{\lambda }$ the ratio between absorption and total extinction, a large variability is observed in shorter wavelengths, which is consistent with the variability in *k*_λ_. In the visible range at 532 nm, the BrC exhibited minimal values (∼0.897), which were higher than those observed for fresh biomass-burning and O_3_-oxidized, but lower than those for NO_3_-oxidized, emphasizing the absorption capabilities of BrC in this wavelength. At 660 nm, they all agree with $\overset{®}{\omega_{\lambda }\;}$ = 1.0, and then the particle is nonabsorbing. The other two parameters (*g*_λ_andβ_λ_) are somehow related to the forward-to-backscattering ratio, and the observed values of *g*_λ_ > 0.6 plus the low values of β_λ_ < 0.2 suggest a strong predominance of forward scattering. The spectral dependences, although still present, are not as remarked as for *k*_λ_ and $\overset{®}{\omega_{\lambda }\;}$ since *g*_λ_ and β_λ_ also depend on the real part of refractive index, which, as discussed before, was not so variable with wavelength. Consequently, the findings presented herein underscore the necessity of accurately determining the optical properties of particles generated by different SOA processes.

Estimates of RFE_λ_ for the four different particle schemes are given in Table 2. RFE_λ_ is defined as the perturbation of the solar flux caused by the presence of aerosols relative to the absence of aerosols (clear sky, no aerosols).^26,27^ A positive RFE_λ_ implies warming, while a negative RFE_λ_ implies cooling. In the RFE_λ_ estimates, we used *m*_λ_, ${\mathrm{\omega ̅}}_{\lambda },g_{\lambda }$ , and β_λ_ for biomass burning wood tar aerosol (fresh, O_3_-oxidized, and NO_3_-oxidized) and those obtained in our study (Table 1).

The results presented in Table 2 demonstrate significant variations in the RFE_λ_ at the top of the atmosphere (TOA), particularly at shorter wavelengths, depending on the type of particle. At 405 nm, for instance, RFE_λ_ values range from a moderate cooling effect for O_3_ oxidation (−2.49 W·AOD^–1^·m^–2^) to a strong warming effect for NO_3_ oxidation (14.89 W·AOD^–1^·m^–2^), with BrC showing a modest warming (1.56 ± 1.88 W·AOD^–1^·m^–2^). This emphasizes the pivotal function of chemical aging pathways in determining aerosol radiative behavior within the UV spectral region. At 532 nm, all systems except NO_3_ exhibit net cooling, with RFE_λ_ values of −19.28 (O_3_), −14.83 ± 1.91 (BrC), and −20.58 W·AOD^–1^·m^–2^ (fresh particles). Conversely, the NO_3_-oxidized system exhibited a modest warming effect (1.12 W·AOD^–1^·m^–2^), suggesting the persistence of light-absorbing species at green wavelengths. At longer wavelengths (650 and 660 nm), all reported RFE_λ_ values become increasingly negative, reflecting a dominant cooling effect. For instance, at 650 nm, fresh, and NO_3_-aged particles demonstrate robust cooling (−24.29 and −23.11 W·AOD^–1^·m^–2^, respectively), while the O_3_-aged particles reach −28.95 W·AOD^–1^·m^–2^. At 660 nm, only BrC RFE_λ_ is reported (−19.48 ± 0.02 W·AOD^–1^·m^–2^), but it continues the trend of increasing net cooling with wavelength. It has been established that, in general, all particle types exhibit decreasing RFE_λ_ (more negative values) as the wavelength increases. However, the rate and magnitude of this variation are contingent on the specific oxidation pathway and the resulting chemical composition.

The results of this study underscore the significance of possessing a comprehensive understanding of the optical properties of the diverse carbonaceous particles that can be produced in the atmosphere by different SOAs, as these optical properties ultimately determine the radiative forcing impact of these particles. A comprehensive analysis of the direct radiative effects of absorbing organic aerosols must consider the size distribution and atmospheric abundance of these particles. However, this study does not cover such an analysis. An assessment of BrC particles with different wavelength dependences and values of *n*_λ_ and *k*_λ_ has been conducted in RFE_λ_. The outcome of this evaluation is contingent on the various formation process pathways. It has been demonstrated that the magnitude of warming is typically greater at shorter wavelengths, while the magnitude of cooling increases with wavelength. Nevertheless, the discrepancies in the pathway formation indicate the intricacy of the problem and the necessity of a more profound comprehension of the formation process to model BrC in the atmosphere. Consequently, enhanced characterization of BrC formation pathways is imperative for more precise climate modeling, as the impact of absorbing organic compounds on climate varies depending on their provenance. Recent reanalysis models that incorporate aerosol modules, such as MERRA-2 and CAMS, do not include BrC as an aerosol type.^28,29^ It has been shown that these particles indeed include black carbon and organic carbon species. However, the optical properties for these species are based on the optical properties of aerosols and clouds package, whose combinations cannot reproduce the spectral dependencies in the imaginary refractive index obtained in this work, which will require future developments to reproduce the high absorption in the UV that has been shown here to be critical for BrC particles.^30^ Therefore, the results and discussions presented here serve to advance the current state of knowledge of the impact of BrC on the climate system.

## Conclusions

This study presents the first analysis of the evolution of the optical and microphysical properties of FeCl_3_ when mixed with fumaric acid within a brown carbon (BrC) formation pathway by using a single-particle analysis platform. The initial experiment showed that fumaric acid significantly alters the physicochemical properties of aqueous FeCl_3_ microdroplets, particularly during hygroscopic cycling. The irreversible increase in particle size and real refractive index following a dehydration–rehydration cycle, despite RH returning to near-initial levels, highlights the complex interplay between chemical composition, water content, and optical behavior. The study resolved dynamic transformations that would otherwise be obscured by ensemble-based analyses by applying phase function fitting based on the Lorenz–Mie theory. A subsequent experiment confirmed the reliability of the experimental results and the accuracy of the complex refractive indices obtained from both CRDS and phase function (PF) measurements by showing that the retrieved particle radii were consistent across all phase function data sets (wavelengths of 473, 532, and 660 nm). The strong agreement at a 532 nm wavelength for the real and imaginary components of the refractive index confirms the robustness of the methods. Notably, the imaginary part exhibits pronounced spectral dependence, with absorption at a wavelength of 405 nm being an order of magnitude greater than at a 532 nm wavelength. This indicates enhanced scattering and absorption in the ultraviolet spectrum. A similar, albeit less pronounced, spectral trend is observed for the real part of the refractive index. Overall, these results emphasize the importance of developing a more comprehensive understanding of the optical properties of BrC formed through various SOA pathways. Current climate models often fail to adequately represent BrC, resulting in an inability to capture its strong UV absorption. Incorporating detailed BrC formation mechanisms and wavelength-dependent optical data into future modeling efforts is therefore essential if we are to improve the accuracy of predictions regarding the interaction between aerosols and radiation and of overall climate projections.

## Materials and Methods

### Chemicals and Solution Preparation

All chemicals were utilized in their original state, without undergoing any additional purification processes. Fumaric acid (99, CAS: 110-17-8, Sigma-Aldrich) and iron­(III) chloride hexahydrate (97, CAS: 10025-77-1, Sigma-Aldrich) were utilized in the experiment. Aqueous-phase solutions were prepared by means of the dissolution of the chemicals in Milli-Q water (18.5 MΩ·cm, pH ∼ 6). A series of reaction solutions were formulated to ascertain the optimal reactant concentrations for the trapping of a single microdroplet. To prepare a reaction solution containing 0.03% Fe (w/w), fumaric acid was first dissolved in 25 mL of Milli-Q water. Subsequently, FeCl_3_ was added to achieve the desired final concentration, while maintaining an approximate Fe-to-fumaric acid ratio of 1:2. The pH of the resulting solution was confirmed to be acidic, with a reading of approximately 3 ± 1.

### Experimental Setup

Figure 6 presents a schematic diagram of the PET and CRDS platforms along with an artistic representation of the trapping scheme. The PET system is accommodated within a custom-built chamber and functions at atmospheric pressure and ambient temperature, with all measurements performed at a temperature of 295.0 ± 0.1 K. The trapping platform comprises two conical electrodes separated by a 1.5 mm gap and enclosed within grounded cylindrical shields with a diameter of 2 cm and a length of 3 cm. An alternating current (AC) voltage signal is applied to the conical electrodes, with an operational amplitude ranging between 1 kVpp and 2 kVpp and a frequency range of 1–2 kHz. In such conditions, the PET has the capacity to trap particles with radii ranging from 700 nm to a few micrometers. RH inside the trapping cell was monitored using a capacitance probe (Honeywell HIH-3610 Series sensor) located 1 cm from the droplet trapping location. The capacitance probe’s manufacturer-stated absolute accuracy is ±2% RH. Two mass flow controllers independently manage the dry and wet N_2_ streams, with the mixing ratio adjusted to achieve the desired RH. The resulting gas mixture is introduced from the top of the chamber, with a total combined flow rate of 20 cm^3^/min. It is posited that the minimal aerodynamic forces exerted on the trapped particles ensure that their position remains stable throughout the experiment. Unless otherwise indicated, the uncertainties reported as “±” throughout the article correspond to standard deviations (SD) obtained from repeated measurements during each experimental period and therefore primarily reflect measurement reproducibility (temporal stability). Values presented in Tables 1 and 2, by contrast, correspond to mean ± SD across independent droplets (interdroplet variability); the number of droplets (*n*) is given in each table caption.

**Figure 6:**
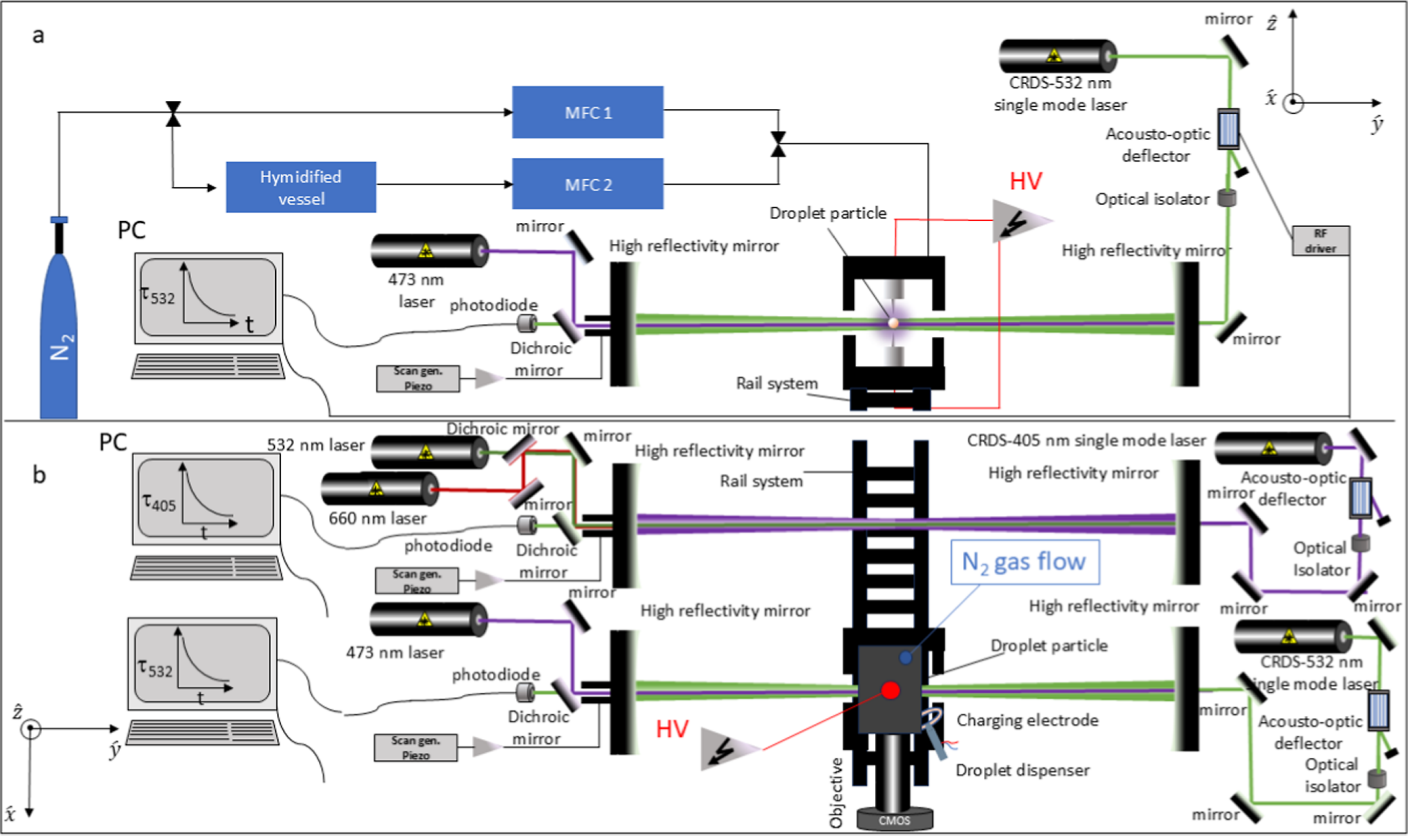
(a) Scheme of the dual CRDS setup, RH control system, and the PET seen from the front and (b) seen from the top.

In this study, a novel CRDS setup with two independent, parallel systems positioned 10 cm apart along axial axes, operating at wavelengths of 405 and 532 nm, is utilized. The ring-down time, denoted by τ_λ_ is measured by these systems. The ring-down time is defined as the time required for the transmitted light intensity to decay to 1/e of its initial value. Further details pertaining to the methodological specifics of CRDS can be found in the Supporting Information.

### Experimental Procedure 1: Hydration–Dehydration Cycle

In the initial experiment, a single microdroplet of the FeCl_3_–fumaric acid solution was captured and exposed to a complete hygroscopic cycle under controlled environmental conditions. The solution was kept in the dark, and measurements began approximately 5 min after the addition of FeCl_3_. The solution was transferred to a microdroplet dispenser (Microfab, MF-ABP-01) and sprayed onto PET, thereby initiating the experimental process. The RH inside the trapping chamber was maintained at 80% for 23 min before subsequent adjustments. Subsequently, the RH decreased to approximately 50% and then increased to approximately 78%, completing a full hydration–dehydration cycle within approximately 45 min. This experiment was repeated on 11 independent droplets.

### Experimental Procedure 2: BrC Aging Simulation

In the second experiment, changes in the optical properties due to aging were assessed. To this end, the FeCl_3_–fumaric acid solution was subjected to 24 h of chemical aging under dark conditions in the bulk phase, to simulate the chemical aging of BrC. Following filtration using ultrapure water, a single aged droplet was levitated and analyzed for 20 min under constant RH conditions (∼75%). As outlined in the optical measurements and retrieval of properties section, the phase function and extinction cross-section measurements were conducted in a sequential manner at four distinct wavelengths. This experiment was repeated on 5 independent droplets.

### Optical Measurements and Retrieval of Properties

Measurements are made of the time constants for exponential decay of light from the desired TEM_00_ mode when the cavity is empty (τ_0_,_λ_) and when it contains a particle (τ_λ_). The particle extinction cross-section (σ_ext_,_λ_) is derived from the difference in the reciprocals of these ring-down times, as described by the following equation^31^

1 $$\sigma_{ext,\lambda }=\frac{\pi w_{0,\lambda }^{2}L}{2c}\left(\frac{1}{\tau_{\lambda }}-\frac{1}{\tau_{0,\lambda }}\right)$$

where *w*_0_,_λ_ is the theoretical beam waist in the geometric center of the cavity, *L* is the length of the cavity, and *c* is the speed of light.

A CMOS camera (Thorlabs, model DCC1546M) is used to collect the elastic light scattering of the trapped particle. The camera is coupled to a 20× long working distance lens (Mitutoyo) with a numerical aperture (NA) of 0.42, positioned at 90° relative to the CRDS laser beam. The two-dimensional (2D) image, often referred to as the particle phase function, is transformed into one-dimensional (1D) spectra by averaging each column of the image over scattering angles ranging from 69 to 111 degrees. The resulting 1D phase function is then compared to a library of phase functions calculated with Mie theory for a physically plausible range of radii and *m*_λ_. Pearson’s correlation coefficient (C) analysis is used to compare the calculated and experimental spectra to determine the best fit.^32^ Most information about procedure fitting details can be found in the Supporting Information.

The same trapped particle is moved between the CRDS systems to perform sequential measurements, ensuring consistent comparison across wavelengths. The movement of the PET on the rail during the different stages of the experiment corresponds to the x̂-axis shown in Figure 6. During the operation of the 405-CRDS system, a laser beam (532 nm wavelength, Gaussian) is used to illuminate the trapped particle for phase function measurements, while the extinction cross section is measured at 405 nm. Subsequently, while maintaining the same particle in a state of capture, the PET is moved along the rail to the 532-CRDS system. In this configuration, a second laser beam at a wavelength of 473 nm is directed onto the particle to carry out phase function measurements. Concurrently, the extinction cross-section is measured at a wavelength of 532 nm. Finally, the PET is returned to the 405-CRDS system to collect phase function measurements at 660 nm. For the sake of clarity, the real and imaginary parts of the refractive index are denoted with subscripts PF and CRDS, respectively, in order to distinguish between fittings derived from phase function (*n*_PF_,_λ_, *k*_PF_,_λ_) and those obtained from CRDS measurements (*n*_CRDS_,_λ_, *k*_CRDS_,_λ_), respectively.

For each phase function measurement, *r* and *m*_λ_ are obtained. These retrieved values are then utilized in an inversion scheme to obtain *r* and *m*_λ_ at the correlative wavelength when extinction cross-section measurements are performed. The procedure to do that consists of fitting the complete set of measured extinction cross-section data, σ_ext_,_1Hz_, to the theoretical, σ_ext_, calculated using Mie theory, through an iterative process that minimizes the reduced cumulative fractional difference (CFD_R_), defined as

2 $${CFD}_{R}=\frac{1}{N}\sum_{i=1}^{N}\frac{\left|\sigma_{ext,\lambda ,Mie}-\sigma_{ext,\lambda ,1Hz}\right|}{\sigma_{ext,\lambda ,1Hz}}$$

where σ_ext_, Mie is the theoretical extinction cross-section, and *N* represents the number of different particle radii considered. A detailed description of the methodology is provided in ref 25.

### Calculation of Optical Properties and Radiative Forcing Efficiency (RFE_λ_)

To assess the atmospheric implications of BrC particles formed through different SOA processes, we calculated their key optical properties and estimated their RFE_λ_. The optical properties analyzed include the complex refractive index (*m* = *n*_λ_ + *ik*_λ_), the single scattering albedo ( $\overset{®}{\omega_{\lambda }\;}$ ), the asymmetry parameter (*g*_λ_) and the backscattering fraction (β_λ_). These parameters were derived using Mie theory, assuming spherical particles.

Experimentally values of *n*_λ_ and *k*_λ_ were obtained for the BrC particles generated in this study and compared with literature data for other BrC types from biomass burning wood tar aerosols under fresh and oxidized conditions (with NO_3_ and O_3_), as reported in ref 11 For the sake of consistency, only the mean values were used in the calculations; standard deviations were not included.

Since detailed number size distributions for BrC are largely unknown and highly variable depending on the source and environment, we adopted a simplified approach by fixing the particle radius to the effective value observed in our study (*r* = 1170 nm). The size parameter (*x*) was computed as

3 $$x=\frac{2\cdot \pi \cdot r}{\lambda }$$

where *r* is the effective particle radius, and λ is the wavelength.

The RFE_λ_ approach normalizes flux variations by aerosol optical depth (AOD_λ_), providing a per-particle efficiency independent of the total aerosol burden. Such an approach has been used in previous studies of RFE_λ_.^33–38^ For simplicity, we use the same equations and base-level assumptions as those in ref 34

4 $${RFE}_{\lambda }=\frac{\Delta F}{{AOD}_{\lambda }}=SD\left(1-A_{cld}\right)T_{atm}^{2}{\left(1-R_{sfc}\right)}^{2}\left[2R_{sfc}\frac{1-\overset{®}{\omega_{\lambda }\;}}{{\left(1-R_{sfc}\right)}^{2}}-\beta_{\lambda }\overset{®}{\omega_{\lambda }\;}\right]$$

where *S* is the solar constant (set to 1370 W m^–2^) at the top of the atmosphere. For the rest of the parameters, we assumed the standard conditions of a continental region: *D* is the fractional day length (set to 0.5), *A*_cld_ is the fractional cloud cover (set to 0.61), *T*_atm_ is the solar atmospheric transmittance (set to 0.76), and *R*_sfc_ is the surface albedo (set to 0.15). β_λ_ is a function of the hemispheric backscattering fraction *b*_λ_, defined as the ratio of backscattering efficiency to total scattering efficiency, and $\overset{®}{\omega_{\lambda }\;}$ is the single scattering albedo caused by a uniform and optically thin aerosol layer. The parameter β_λ_ was calculated from the Henyey–Greenstein phase function

5 $$\beta_{\lambda }=0.082+1.85\cdot b-2.97\cdot {b_{\lambda }}^{2}$$

whereas *b*_λ_ was derived from *g*_λ_ through the equation given in ref 39

6 $$b_{\lambda }=\frac{1-{g_{\lambda }}^{2}}{2g_{\lambda }}\left(\frac{1}{\sqrt{1+{g_{\lambda }}^{2}}}-\frac{1}{\sqrt{1+g_{\lambda }}}\right)$$

This approach assumes a thin aerosol layer in the lower troposphere under continental conditions. Calculations were performed for the optical properties corresponding to different particle formation pathways, including biomass burning wood tar aerosols (fresh, O_3_-oxidized, and NO_3_-oxidized) and BrC particles from this study.

## Supplementary Material



## Notes

Detailed materials, experimental methods, modeling with Supporting Figures S1 and S2, and data from a representative control experiment for a levitated aqueous microdroplet containing FeCl_3_ (no fumaric acid) (PDF)
