# Supplementary material for: Secondary Brown Carbon Formed by a Microreactor of a Levitated Aqueous Fe (III) Droplet with Fumaric Acid
Source: ACS Photonics. 2025 Dec 25;13(1):67–79. doi: 10.1021/acsphotonics.5c01691 (PMC12784408; doi:10.1021/acsphotonics.5c01691)
Supplement: Supplementary file 1 [file ph5c01691_si_001.pdf]

Supporting Information

Secondary brown carbon formed by a microreactor of levitated aqueous Fe (III) droplet with fumaric acid

Gema Sánchez-Jiménez[‡§], Hind A. Al-Abadleh[ξ], Daniel Pérez-Ramírez[‡§], Lucas Alados-Arboledas[‡§], Francisco José Olmo-Reyes[‡§], and Antonio Valenzuela[‡ §*]

[‡] Andalusian Institute for Earth System Research (IISTA-CEAMA), Granada, 18006, Spain

[§] Department of Applied Physics, University of Granada, Granada, 18071, Spain

[ξ] Department of Earth, Environmental and Resource Sciences, University of Texas at El Paso, 500 West University Avenue, El Paso, Texas 79902, United States.

*Corresponding author email address: avalenzuela@ugr.es

ACS Photonics

Prepared: November 19, 2025

Supporting Information (6 pages)

Details on Methods and Calculations S2
Description of experiment control S3
Measurements of ring down time S3

Details on Methods and Calculations:

Cavity ring down spectroscopy setup procedure. The particle is precisely positioned at the central maximum of the TEM_{00} Gaussian mode of the cavity beam by carefully adjusting the PET with a 3-D micrometric translation stage. As shown in Figure 1a (Manuscript), the coordinate system (x, y, z) indicates the three degrees of freedom available for particle translating. The PET is mounted on a rail which allows controlled transverse and axial movement relative to the optical axis of each cavity. One mirror of each CRDS cavity is mounted on a Piezomechanik piezo ring actuator, which is used to sweep the cavity into resonance with the laser. The actuator is driven with a triangular waveform (10 V amplitude, 2–10 Hz frequency), allowing the cavity to build up on a single longitudinal mode. The laser beam is coupled into the cavity through an acousto-optical deflector (AOD), which focuses and modulates the beam. Ring-down events are initiated by interrupting the input beam via the AOD. Measurements are made of the time constants for exponential decay of light from the TEM_{00} mode when the cavity is empty ($\tau_{0,\lambda}$) and when it contains particle (τ_{λ}). For alignment, we use the cavity ring-down time (τ_{λ}) as an overlap metric. The PET is translated iteratively along the x, y, and z axes until τ_{λ} and its shot-to-shot variability reach a minimum. Under optimal overlap conditions, the standard deviation of τ_{λ} is typically below 0.25 µs. This criterion confirms that the trapped particle is fully overlapped with the TEM_{00} Gaussian mode and positioned at the central standing-wave. Once this condition is achieved, the particle remains fixed throughout the entire measurement sequence in that cavity. When the PET is moved from one CRDS system to another, the same translation and τ_{λ}-minimization procedure is repeated to re-establish the particle at the geometric centre of the second cavity. This ensures that in both cavities the particle occupies the same relative position within the standing-wave field. The two CRDS systems are 1 m optical cavities with mirror radii of curvature of 1 m, and the mirrors have reflectivity of >99.987% at 532 nm and >99.985% at 405 nm. The estimated beam waist ($w_{0,\lambda}$) at the focal point and the ring down time for the empty cavity ($\tau_{0,\lambda}$) are ~291 µm and 18.79 ± 0.22 µs, respectively, for 532-CRDS system, being ~271 µm and 16.80 ± 0.23 µs, respectively, for 405-CRDS system. To reduce the contributions of airborne dust particles to light extinction and to prevent the mirrors getting dirty, nitrogen gas flows are directed across the faces of the cavity mirrors through that extend to a trapping cell at the center of the cavity.[1] The difference in the reciprocals of the ring-down times is proportional to the $\sigma_{ext,\lambda}$ given for the following equation:

$$\sigma_{ext,\lambda} = \frac{\pi w_{0,\lambda}^2 L}{2c}\left(\frac{1}{\tau_\lambda} - \frac{1}{\tau_{0,\lambda}}\right) \quad (1)$$

where L is the length of the cavity and c is the speed of light.

The light trapped within an optical cavity forms a standing wave along the cavity axis, which arise from the interference of two counter-propagating plane waves. Miller and Orr-Ewing defined an equation which modifies the Mie extinction cross-section to account correctly for the effect of the standing wave structure on measurements in a ring-down cavity [2]. Mie $\sigma_{ext,\lambda}$ values depending on the position of particle in the standing wave with limits established in anti-node ($ky_0 = 0$) and node ($ky_0 = \frac{\pi}{2}$) positions. Particle centred half-way between a node and an anti-node ($ky_0 = \frac{\pi}{4}$) agrees exactly with the Mie theory prediction of $\sigma_{ext,\lambda}$. The equivalence of the Cavity Standing Wave Generalized Lorenz-Mie theory (CSW-GLMT) predicted CRDS measurements of $\sigma_{ext,\lambda}$ for

the case $ky_0 = \frac{\pi}{4}$ and the Mie theory predicted $\sigma_{ext,\lambda}$ for a traveling wave were exploited in ref [3]. Therefore, although the sampling rate in our experiment was greater than 5 Hz, since the timescales for changes in optical properties such as size and refractive index were larger than 1 s, we averaged our measurements at 1 Hz intervals. That way our $\sigma_{ext,\lambda,1Hz}$ values converged to the Mie theory limit.

Elastic scattering setup. The PET is located inside a custom-made chamber operating under atmospheric pressure with all measurements performed around 295 K. This trap can suspend single microdroplets in the radius range between 700 nm up to a few micrometers. The RH inside the PET chamber is controlled by two MKS mass flow controllers, one for dry N_2 gas and the other for wet N_2 gas and monitored using a capacitance probe (Honeywell). The air mixture is introduced from the top of the PET cell at flow rates up to 20 cm^3 min^{-1}. The aerodynamic force on the microdroplet is small, so it does not significantly affect its position. The r was determined using a rigorous two-step fitting protocol, which compared experimental phase functions (PFs) to a simulated library generated through Lorentz-Mie theory across a plausible range of radii. This approach incorporated variability both the n_λ and k_λ at a specific wavelength. Initially, the value of $k_{PF,\lambda}$ was fixed at zero to identify the optimal r and $n_{PF,\lambda}$ by maximizing C between the experimental and theoretical PFs. Once the optimal $n_{PF,\lambda}$was identified, it was held constant while r and $k_{PF,\lambda}$ were iteratively adjusted to refine the fit and maximize C. This comprehensive methodology allowed for precise identification of $n_{PF,\lambda}$, $k_{PF,\lambda}$, and r, facilitating a robust characterization of the droplet's optical and microphysical properties. The uncertainties associated with these parameters, particularly the r, were rigorously assessed in ref 4. Parameters from experimental phase-function fittings were binned in 2 % RH intervals, chosen to be consistent with the manufacturer-stated absolute accuracy of the capacitance probe (±2 % RH). Repeated measurements of parameters within each RH bin were averaged, and the standard deviation (SD) was calculated to quantify reproducibility. Note that SD values (reported throughout as mean ± SD) describe the spread of repeated measurements, while the probe's absolute accuracy (±2 % RH) represents an additional systematic uncertainty in the true RH value [4,5].

Description of experiment control:

Control experiments were conducted using microdroplets of FeCl3 at a concentration of 0.03 wt% Fe, to establish a baseline for comparison with microdroplets from reaction solutions containing fumaric acid. The FeCl3 microdroplets were trapped in suspension at 80% relative humidity (RH) for 1.5 hours and the scattered light was recorded. At 2200 seconds, dehydration of microdroplets was initiated by reducing the RH to 50%. After 1100 seconds, hydration was initiated, returning to 80% RH at the same rate. These experiments were repeated 3–5 times to ensure reproducibility.

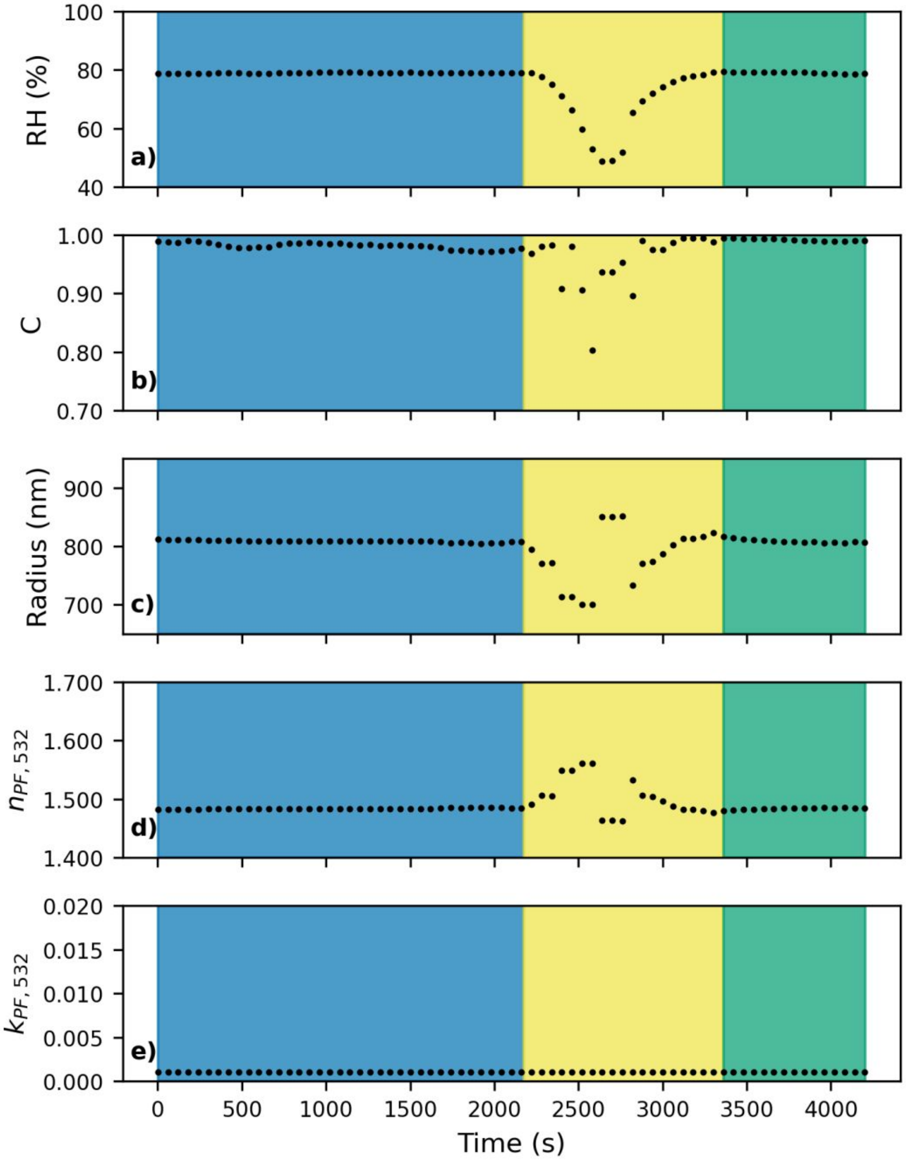

Figure S1. Data from a representative control experiment of evolution of the optical and microphysical properties of a microdroplet of $FeCl_3$ (no fumaric acid). The plot showing: a) the values in measured %RH, b) Pearson's correlation coefficient (C), c) fitting radius form Mie theory, d) $n_{PF,532}$ and e) $k_{PF,532}$ with increasing reaction time. The shaded regions correspond to the initial RH (blue), the dehydration and hydration cycle (yellow), and the final RH (green).

Measurements of ring down time:

A $FeCl_3$–fumaric acid microdroplet was trapped and levitated in the PET. Initially, the PET was positioned in the 405-CRDS system, where ring-down time measurements at 405 nm were recorded (Figure S2a). The same particle was then transferred along the rail to the 532-CRDS system, where ring-down time measurements at 532 nm were obtained (Figure S2b).

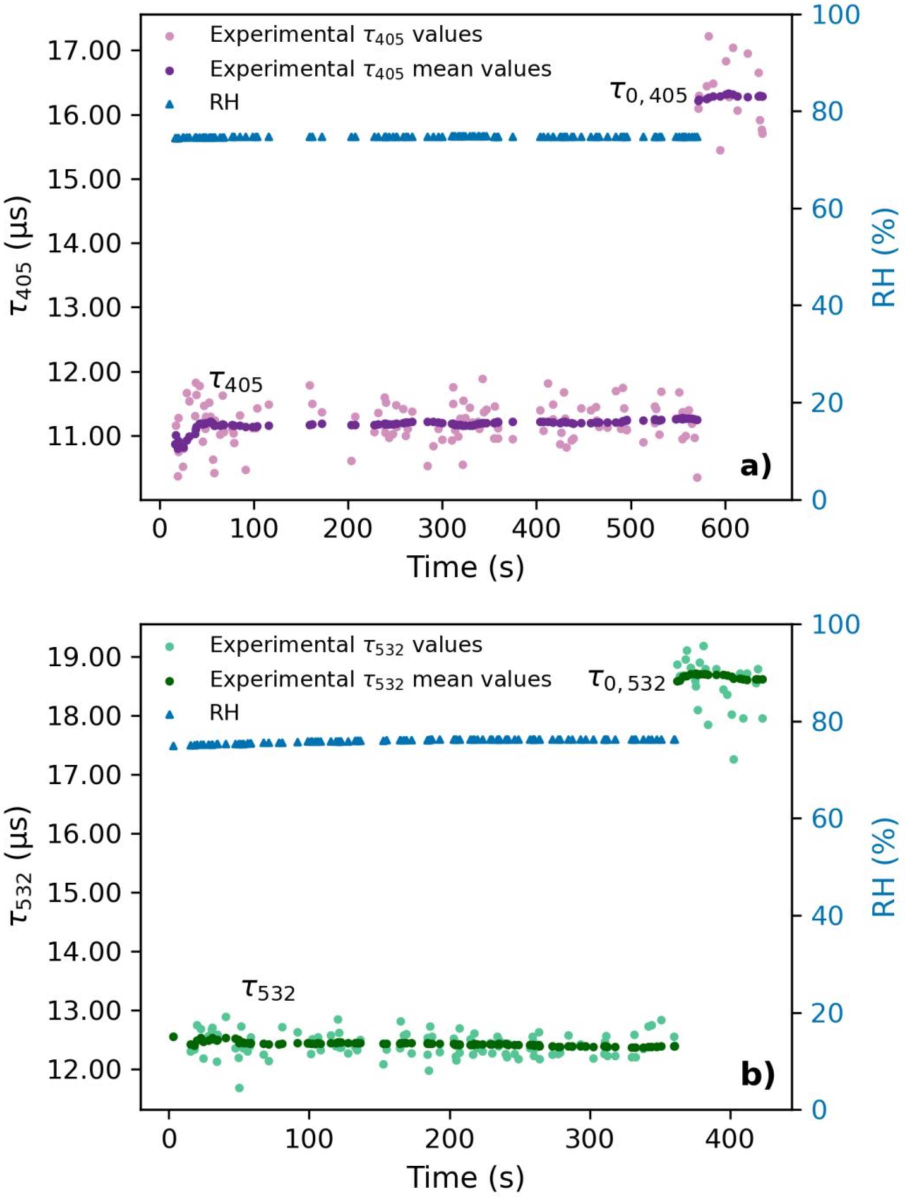

Figure S2. Representation of the experimental ring down time measurements at 405 and 532 nm of microdroplets of $FeCl_3$-fumaric acid system, which were stored in the dark for 24 h to simulate ageing. The data shown: a) τ_{405} and $\tau_{0,405}$ obtained for CRDS-405, along with their mean values and b) τ_{532} and $\tau_{0,532}$ obtained from CRDS-532, including their mean values. Blue dots correspond to RH.

(1) Valenzuela, A.; Bazo, E.; Rica, R. A.; Alados-Arboledas, L.; Olmo-Reyes, F. J. Electrodynamic single-particle trap integrated into double-cavity ring-down spectroscopy for light extinction. *J. Aerosol Sci.* **2024**, *175*, 106292. DOI: 10.1016/j.jaerosci.2023.106292.

(2) Miller, J. L.; Orr-Ewing, A. J. Cavity ring-down spectroscopy measurement of single aerosol particle extinction. II. Extinction of light by an aerosol particle in an optical cavity excited by a cw laser. *J. Chem. Phys.* **2007**, *126*, 174307. DOI: 10.1063/1.2723736.

(3) Cotterell, M. I.; Knight, J. W.; Reid, J. P.; Orr-Ewing, A. J. Accurate measurement of the optical properties of single aerosol particles using cavity ring-down spectroscopy. *J. Phys. Chem. A* **2022**, *126*, 2619–2631. DOI: 10.1021/acs.jpca.2c01246.

(4) Valenzuela, A.; Rica, R. A.; Olmo-Reyes, F. J.; Alados-Arboledas, L. Testing a Paul trap through determining the evaporation rate of levitated single semi-volatile organic droplets. *Opt. Express* **2020**, *28*, 34812. DOI: 10.1364/OE.410590.

(5) Cai, C.; Marsh, A.; Zhang, Y.; Reid, J. P. Group contribution approach to predict the refractive index of pure organic components in ambient organic aerosol. *Environ. Sci. Technol.* **2017**, *51*, 9683–9690. DOI: 10.1021/acs.est.7b01924.
